# Financial barriers for medical students attending ophthalmology conferences: an analysis of registration fees

**DOI:** 10.3389/fmed.2025.1708446

**Published:** 2025-11-19

**Authors:** David Hutchins, Meghan K. Berkenstock

**Affiliations:** 1 Drexel University College of Medicine, Philadelphia, PA, United States; 2 The Wilmer Eye Institute, Division of Ocular Immunology, The Johns Hopkins University School of Medicine, Baltimore, MD, United States

**Keywords:** medical students, conference participation, financial barriers, ophthalmology, academic ophthalmology

## Abstract

**Purpose:**

Ophthalmology conferences are vital for knowledge exchange, networking, and professional development in ophthalmology. However, financial barriers may limit medical students’ participation. This study assesses the costs associated with attending major United States conferences and examines disparities in funding availability based on institutional and conference-specific factors.

**Design:**

A descriptive cross-sectional analysis was conducted using publicly available data.

**Subjects, participants, or controls:**

Not applicable; this study analyzed publicly available data on conferences and institutions.

**Methods:**

A cross-sectional analysis was conducted to assess conference-related expenses from January to December 2024. Data on registration fees, travel and accommodation costs, and available travel grants were collected from official conference websites and the publicly available websites of U.S. allopathic medical schools. Data were organized and analyzed descriptively using Microsoft Excel (Microsoft Corporation, Redmond, WA, USA), with comparisons made between general and subspecialty conferences and between virtual and in-person participation to illustrate cost variability.

**Results:**

Conference registration fees varied substantially, with a mean in-person registration cost of $670.83 (SD = $391.65), ranging from $0 to $1,100. Virtual fees averaged $541.67 (SD = $166.46), ranging from $400 to $725. Student discounts were inconsistently offered, and some conferences provided no financial relief. Travel and lodging added a significant expense. Institutional travel stipends ranged from $0 to $2,500 (mean = $428.33, SD = $144.83), often falling short of total costs.

**Conclusion:**

Medical students encounter considerable out-of-pocket costs when participating in ophthalmology conferences. Addressing these financial barriers through expanded travel funding, standardized student discounts, and reduced registration fees could enhance equity in access to professional development and foster greater inclusion within the field.

## Introduction

The rising costs of professional education and training are a significant concern for medical students and professionals (1). As in other specialties, conferences in ophthalmology serve as essential platforms for knowledge exchange, networking, and exposure to the latest advancements (2). However, the financial burden of attending these events might be a barrier, particularly for medical students.

Prior research has highlighted the increasing costs of medical conferences across various specialties (3). Studies have shown that registration fees, travel expenses, and accommodation costs are significant deterrents to conference participation for students and residents (4). In fields such as ophthalmology, where research presentations and networking play a crucial role in securing residency and fellowship positions (5, 6), these financial barriers can disproportionately impact students from lower-income backgrounds (7). At the same time, conference presentations may enhance the match success in competitive specialties such as ophthalmology (8). Some medical schools provide travel stipends for conference attendance (9), but these policies vary widely.

We analyzed the costs associated with attending ophthalmology conferences from 2024 to 2025 and identified potential improvement areas to enhance accessibility. Specifically, we examined registration fees, travel expenses, and the availability of financial assistance through travel grants or stipends.

## Patients and methods

This was a descriptive cross-sectional analysis of conference-related expenses for the largest ophthalmology meetings in the United States. These conferences were defined as those with broad national or international participation. The primary focus was on large national and subspecialty meetings held in the United States. Conferences were selected based on visibility, student participation, and availability of public pricing data. Data were collected on registration fees, travel expenses, possible travel grants offered, and accommodation costs from official conference websites and linked travel resources between January and June 2024, with verification in December 2024. Conference websites were specifically reviewed for registration rates applicable to medical students, and student-specific pricing was recorded when available.

Travel expenses were gathered from each conference website’s average reported travel and hotel costs. When travel stipends were available, their criteria were noted. Hotel data were derived from the conference website, using double-occupancy rates when listed. The annual conferences analyzed include the American Academy of Ophthalmology (AAO), Association for Research in Vision and Ophthalmology (ARVO), American Society of Cataract and Refractive Surgery (ASCRS), American Glaucoma Society (AGS), American Association for Pediatric Ophthalmology and Strabismus (AAPOS), North American Neuro-Ophthalmology Society (NANOS), and Women in Ophthalmology (WIO) Summer Symposium. Non-member early-bird pricing was used for fee comparisons.

Additionally, a comprehensive list of allopathic medical schools was obtained from the Association of American Medical Colleges (AAMC) (10) and Liaison Committee on Medical Education (LCME) databases (accessed March 2025). These sources replaced previously used open-access lists to improve reliability. Medical school websites were reviewed for publicly available information on travel stipends for conference attendance. If a school listed a stipend, the criteria and limits were documented. When calculating average funding across schools, the maximum available amount was assumed. Schools without a listed value or explicit mention of student funding were excluded.

Descriptive analyses summarized cost variations across conferences. Data were organized and analyzed using Microsoft Excel (Microsoft Corporation, Redmond, WA, USA). Simple descriptive comparisons were also made between general and subspecialty conferences, and between virtual and in-person participation, to illustrate cost variability. Median and interquartile ranges (IQRs) were reported to provide a clearer view of central tendencies. A Supplementary Table S1 includes all conference URLs, institutional links, and data collection dates to ensure transparency and reproducibility.

This study was deemed exempt by the Drexel University College of Medicine Institutional Review Board and followed the tenets of the Declaration of Helsinki.

## Results

Conference registration fees varied across different ophthalmology meetings (see Supplementary Table S1 for detailed conference-level data). The mean in-person registration fee was $670.83 (SD = $391.65; range $0–$1,100), whereas the mean virtual participation fee was $541.67 (SD = $166.46; range $400–$725). Hotel accommodations represented an additional major expense, typically $150–$400 per night. Student discounts were inconsistently available – some conferences offered none, while others provided reduced rates beginning at $250. Travel-grant opportunities were similarly inconsistent, ranging from no support to awards fully covering registration costs or providing up to $655 in reimbursement.

Travel stipends from U.S. medical schools also varied widely, ranging from $0 to $2,500 (mean = $428.33, SD = $144.83). Out of the 82 medical schools analyzed, two provided no direct funding (Florida Atlantic University Charles E. Schmidt College of Medicine and Geisinger Commonwealth School of Medicine), directing students instead to external or internal non-travel-specific sources.

**Figure 1:**
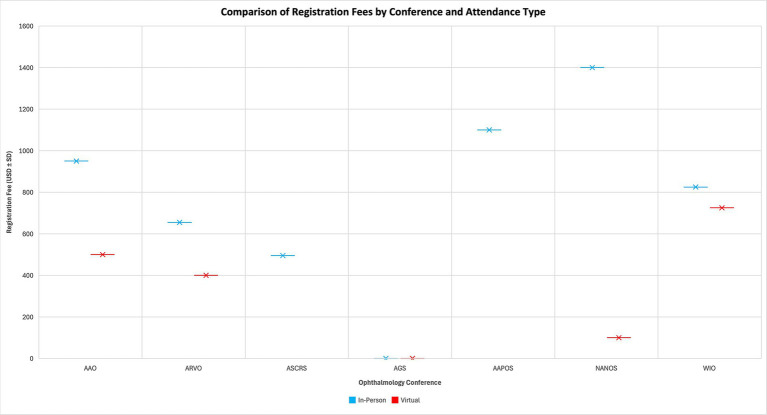
Comparison of registration fees by conference and attendance type (*n* = 7 conferences). In-person registration costs were consistently higher than virtual registration fees across the major U.S. ophthalmology meetings. Values represent the mean cost (USD ± SD).

**Figure 2:**
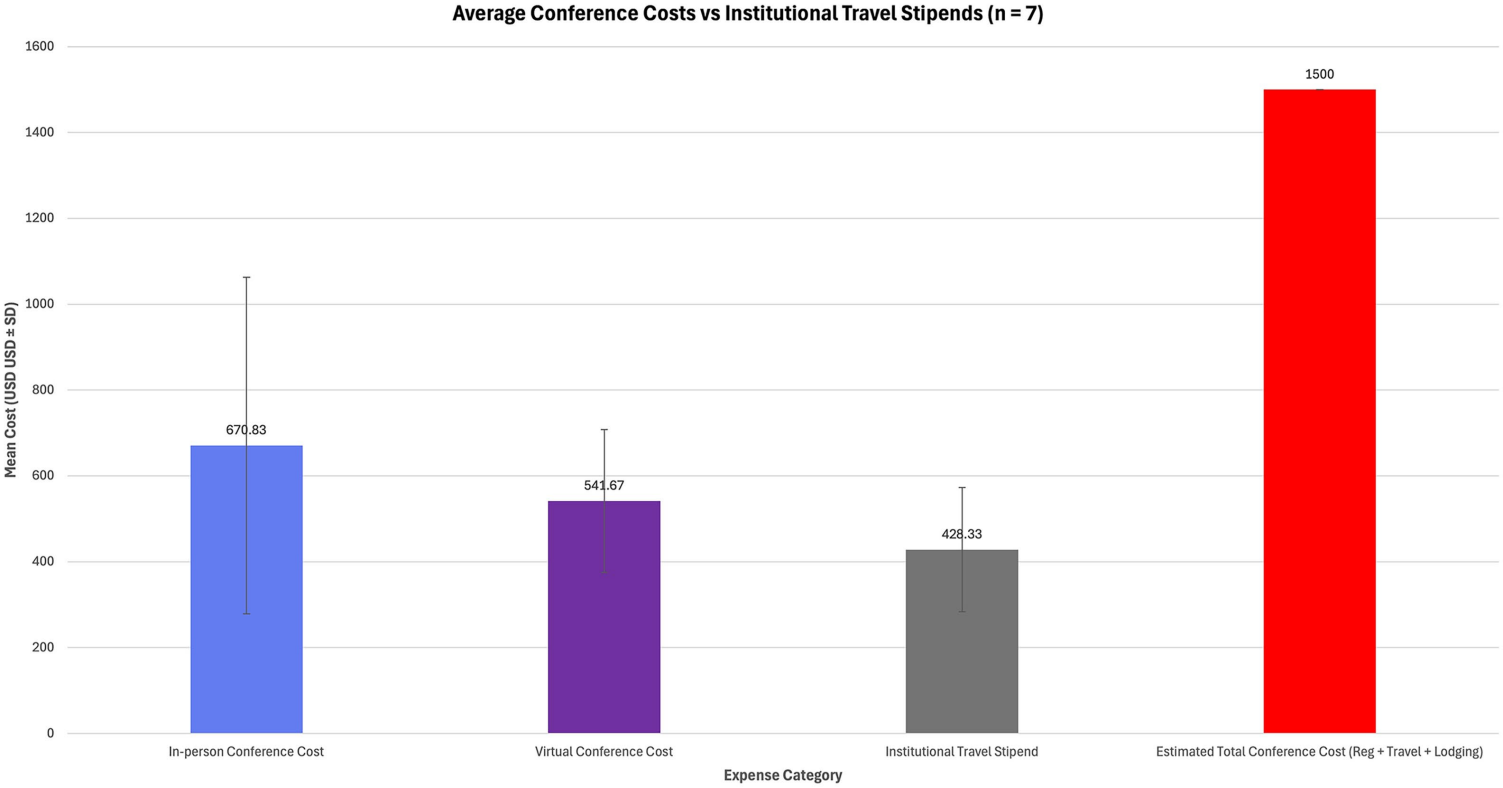
Comparison of average conference-related costs and institutional travel stipends (*n* = 7 conferences). Bars represent mean estimated expenses (USD ± SD) for in-person and virtual attendance, as well as average funding support from medical schools. Total estimated in-person costs exceeded $1,500, while institutional stipends averaged only $428 (SD = 145).

Figure 1 illustrates registration fee variability across *n* = 7 major ophthalmology conferences, demonstrating that in-person attendance consistently incurs higher mean costs (USD ± SD) than virtual participation. Figure 2 compares mean estimated total conference expenses (registration + travel + lodging) with the mean institutional travel stipend, highlighting a persistent funding gap between available support and actual costs.

Among institutions offering direct stipends, approximately 61% (*n* = 50) provided between $400 and $800 per academic year, often with conditions such as presenter status or a once-per-year limit. Higher-end stipends included Emory University School of Medicine (up to $2,500 for international travel), Hackensack Meridian School of Medicine (up to $2,000), and Michigan State University College of Human Medicine (up to $2,000). Lower-range examples include Tufts University School of Medicine ($100–$300) and Loyola University Chicago Stritch School of Medicine (up to $250). Eligibility requirements vary substantially—from first-author presentation mandates (e.g., Oakland University William Beaumont School of Medicine) to small attendance-based reimbursements (e.g., Geisel School of Medicine at Dartmouth). Several schools, such as the Johns Hopkins School of Medicine and the UCLA David Geffen School of Medicine, limited students to a single travel award across their entire training period.

## Discussion

Multiple studies have shown that the cost of medical conferences has steadily increased over the last several years, and many meetings continue to offer limited financial aid options for students. Specific to ophthalmology, our findings highlight the financial burden associated with attending conferences and the rising cost of professional development. As the number of publications per applicant in ophthalmology continues to rise, attending these conferences has become increasingly crucial for networking and research presentations. The mean number of publications of applicants in the San Francisco Match exceeds that of the National Resident Matching Program (NRMP). Even within ophthalmology, the number of publications per applicant has increased in the 5 years between 2017 and 2022 (3 versus 1.5) (11).

Conference participation fosters mentorship, networking, and research exposure. These are key factors that influence professional growth and residency applications. Given the growing emphasis on scholarly output and professional connections, equitable access to these opportunities is essential. Recent changes to the Step 1 exam, which has transitioned to a binary pass/fail system, have removed an objective data point for applicant comparison and increased reliance on Step 2 performance, research productivity, and other non-standardized metrics (12). These shifts may disadvantage certain groups, including international medical graduates and osteopathic applicants, who have faced additional barriers such as lower match rates (45% in 2022 and 32% in 2023) and the loss of dedicated residency positions following the ACGME accreditation transition (13–15).

In terms of total financial considerations, a cross-sectional review of 110 national medical specialty societies in the AMA House of Delegates found that only 13 offered free registration to non-member medical students. Among the 97 societies that charged a student registration fee, the median price was $249.50 (IQR, $150.00–$406.25). Our analysis confirms that ophthalmology conference fees are comparable to or exceed those in other medical specialties, with a mean registration fee for in-person attendance of $670.83 (2, 16). While virtual participation offers a cheaper alternative, it lacks the networking and mentorship opportunities that enhance professional value. From an equity and inclusion standpoint, these findings highlight how economic disparities can shape opportunities for career advancement in ophthalmology. Students from lower-income and underrepresented backgrounds may face disproportionate challenges accessing the same professional networks as their peers, underscoring the need for deliberate institutional and organizational efforts to promote equitable access to mentorship and research presentation opportunities.

Although some conferences and medical schools provide travel grants or reduced registration rates, these resources vary widely in amount, eligibility, and transparency. Among institutions offering direct funding, awards are often limited to presenting authors or once per academic year, restricting access for students seeking continued professional engagement. Funding was also more frequently directed toward residents than students (17). Given that both increased research productivity and networking have been associated with higher match rates (18), ensuring financial support for students to attend conferences is essential for promoting equity in training and opportunity.

Potential strategies to improve accessibility include expanding need-based fee waivers, establishing travel-grant programs modeled after ARVO’s student awards, and increasing hybrid or virtual participation options that lower travel and lodging costs. Implementing these interventions could meaningfully reduce financial barriers and promote greater inclusion in ophthalmology education. A key strength of this study is its timely insight into the increasing financial burden faced by medical students seeking to attend ophthalmology conferences. By quantifying registration fees and comparing them to typical student financial resources, this study highlights an important and under-discussed barrier to professional development. Additionally, it reveals disparities in grant availability and travel support offered by U.S. medical schools, drawing attention to inequities that may influence career exposure and advancement in ophthalmology.

However, the study has limitations. Data on stipends and institutional support for conference attendance were obtained from publicly available websites, which may not capture all current opportunities or reflect recent changes. Pricing categories and discount eligibility also vary across organizations, which could contribute to an underestimation or overestimation of financial support. These limitations reflect a broader lack of transparency and standardization in travel-funding policies for medical students. While increased conference participation and research productivity may correlate with improved patient outcomes, these relationships should be viewed as associative rather than causal. Our descriptive cross-sectional design does not permit inference of direct effects, and future longitudinal studies are needed to clarify these pathways.

While the number of medical students entering the San Francisco Match has risen over the past 5 years, the cost of attending ophthalmology conferences has not decreased. Conferences remain vital for presenting research and networking, allowing students to gain mentorship and professional development (19). Resulting publications can further strengthen a student’s residency application and provide a distinguishing factor amidst less objective comparative measures (20). This paper serves as a call to action for medical schools and conference organizers to collaborate in reducing costs and expanding funding for medical student participation. Such changes are essential to foster a more equitable and diverse ophthalmic community.

## Data Availability

The original contributions presented in the study are included in the article/Supplementary material, further inquiries can be directed to the corresponding author.
