# Supplementary material for: Financial barriers for medical students attending ophthalmology conferences: an analysis of registration fees
Source: Front Med (Lausanne). 2025 Nov 19;12:1708446. doi: 10.3389/fmed.2025.1708446 (PMC12672230; doi:10.3389/fmed.2025.1708446)
Supplement: Supplementary file 1 [file Data_Sheet_1.pdf]

Supplementary Table 1. Comparison of 2024 National Ophthalmology Conference Fees and Availability of Student Discounts

Conference	In-Person Fee (USD)	Virtual Fee (USD)	Student Discount Available?	Travel Grant Amount Available	Criteria for Travel Grant
AAO 2024	$950	$500	Yes ($300)	Not offered to medical students or residents	Reimbursement offered to: Board of Trustees, Committee of Secretaries, Councilors, Academy associate secretaries and committee chairs, Academy representatives to outside organizations, AAOE Board members, Young Ophthalmologist Committee, and YO Advocacy Subcommittee
ARVO 2025	$655	$400	Yes ($250)	At least $655	Travel grants cover Annual Meeting

					registration fees for all recipients, and a stipend to help cover travel expenses is included.
ASCRS 2025	$495	Not available	No	No	
AGS 2025	Free	Free	Yes	No	
AAPOS 2025	$1100	Not Available	Yes ($400)	No	
NANOS 2025	$1400	$100*	Yes (350)	No	
WIO 2025	$825	$725	No	No	

American Academy of Ophthalmology (AAO) annual meeting, Association for Research in Vision and Ophthalmology (ARVO), American Society of Cataract and Refractive Surgery (ASCRS), American Glaucoma Society (AGS) Annual Meeting, American Association for Pediatric Ophthalmology and Strabismus (AAPOS) Annual Meeting, North American Neuro-Ophthalmology Society (NANOS), Women in Ophthalmology (WIO)

*Student pricing was available for virtual attendance and differed from other virtual registration fees.
